# miR-25 Promotes Cell Proliferation, Migration, and Invasion of Non-Small-Cell Lung Cancer by Targeting the LATS2/YAP Signaling Pathway

**DOI:** 10.1155/2019/9719723

**Published:** 2019-06-18

**Authors:** Tangwei Wu, Hui Hu, Tianzhu Zhang, Liyuan Jiang, Xiaoyi Li, Shuiyi Liu, Chao Zheng, Ge Yan, Weiqun Chen, Yong Ning, Yong Li, Zhongxin Lu

**Affiliations:** ^1^Department of Medical Laboratory, The Central Hospital of Wuhan, Tongji Medical College, Huazhong University of Science and Technology, Wuhan 430014, China; ^2^School of Laboratory Medicine, Hubei University of Chinese Medicine, Wuhan 430065, China; ^3^Cancer Research Institute of Wuhan, The Central Hospital of Wuhan, Tongji Medical College, Huazhong University of Science and Technology, Wuhan 430014, China; ^4^Department of Central Laboratory, The Central Hospital of Wuhan, Tongji Medical College, Huazhong University of Science and Technology, Wuhan 430014, China; ^5^Key Laboratory for Molecular Diagnosis of Hubei Province, The Central Hospital of Wuhan, Tongji Medical College, Huazhong University of Science and Technology, Wuhan 430014, China; ^6^Department of Cancer Biology, Lerner Research Institute, Cleveland Clinic, Cleveland, OH 44195, USA

## Abstract

Metastasis is the leading cause of high mortality in lung cancer patients, and metastatic lung cancer is difficult to treat. miRNAs are involved in various biological processes of cancer, including metastasis. Our previous studies revealed that miR-25 promoted non-small-cell lung cancer (NSCLC) cell proliferation and suppressed cell apoptosis by directly targeting *TP53* and *MOAP1*. In this work, we further explored the miR-25 expression in NSCLC patients in the Cancer Genome Atlas (TCGA) database and measured the miR-25 expression levels in the tissues of NSCLC patients and cell lines. miR-25 was overexpressed in both NSCLC tissues and cell lines. NSCLC patients who expressed a higher level of miR-25 exhibited worse overall survival than those with a lower level of miR-25. Overexpression of miR-25 enhanced NSCLC cell migration and invasion, while the inhibition of miR-25 exhibited the opposite effects. We identified the large tumor suppressor homology 2 (*LATS2*) as a new target gene of miR-25 in lung cancer. The effects of miR-25 on promoting NSCLC cell migration and invasion were at least partially due to activation of the Hippo signaling pathway. Additionally, miR-25 antagomir inhibited xenograft tumor growth and metastasis by the upregulation of LATS2. Taken together, our findings demonstrate that miR-25 contribute to lung cancer cell proliferation and metastasis by targeting the LATS2/YAP signaling pathway, which implicate miR-25 as a promising therapeutic target for lung cancer metastasis. Given that oxidative stress induces the overexpression of miR-25 and plays a critical role in cancer progression, this study establishes miR-25 as an intermediate between oxidative stress and lung cancer metastasis.

## 1. Introduction

Lung cancer is the leading cause of cancer-related deaths in both developing and developed countries [1, 2]. Based on the diverse pathobiological features, lung cancer can be divided into two major types: non-small-cell lung cancer (NSCLC) and small-cell lung cancer (SCLC). NSCLC accounts for approximately 80% of lung cancer. Despite recent advances in treatments, the overall 5-year survival rate of patients with NSCLC remains less than 10% [3]. When diagnosed, most NSCLC patients are already at the metastatic stage with poor prognosis; metastasis in cancer is a tough in clinical care of NSCLC [4]. Oxidative stress plays important roles in the pathogenesis of lung cancer, including metastasis [5]. Yet the underlying mechanisms remain elusive.

MicroRNAs (miRNAs) are a group of ~22 nt endogenous noncoding small RNAs that play important roles in the pathogenesis of human diseases by targeting the 3′UTR of mRNAs for mRNA degradation or translational repression [6]. miRNAs are involved in a series of tumor biological processes, such as differentiation, proliferation, apoptosis, migration, invasion, and drug resistance [7–9]. Dysregulated miRNAs may act as tumor suppressors or oncogenes (oncomiRs). Combined serum levels of several miRNAs (miR-9-5p, miR-21-5p, and miR-223-3p) with existing tumor biomarkers offer better detection of NSCLC than the tumor biomarkers alone [10]. Functional studies of dysregulated miRNAs in cancer implicate miRNAs as attractive targets for novel therapeutic development [11, 12]. The antimiRs targeted at miR-122 reached the phase II trials for treating hepatitis [12]. The ongoing phase I trial MesomiR 1 (ClinicalTrials.gov NCT02580552) detected a case of partial response after eight weeks of treatment [13]. In addition, the miRagen company has developed an inhibitor for mir-155, MRG-106, for the treatment of hematologic tumor and is completing phase I clinical trials (http://www.miragen.com/clinical-trials/). Thus, further investigations on miRNA involved in tumor development and metastasis will offer new opportunities in cancer therapy.

miR-25, along with miR-93 and miR-106b, is a member of the miR-106b-25 cluster, which is located in the intron 13 of the minichromosome maintenance complex component 7 (*MCM7*) gene on chromosome 7q22.1. miR-25 is dysregulated in many human cancers with various functions [14]. In glioma, miR-25 promotes glioma cells proliferation by targeting CDKN1C [15]. In gastric cancer, miR-25 promotes cancer progression by directly downregulating ERBB2 and is a noninvasive prognosis biomarker [16]. Colorectal cancer-derived exosomal miR-25 is involved in premetastatic niche formation and may be used as a blood-based biomarker for prediction of metastasis [17]. miR-25 principally functions as oncogene, but in some cases, it may be tumor suppressive. In cisplatin-resistant cervical cancer cells, miR-25 reverses epithelial-mesenchymal transition via targeting Sema4C [18]. miR-25 also modulates invasiveness and dissemination of human prostate cancer cells by targeting the proinvasive *α*v- and *α*6-integrins [19].

Our previous studies revealed that miR-25 was elevated in the plasma of NSCLC patients and NSCLC cell lines, and miR-25 directly targeted and downregulated *TP53* and *MOAP1* in lung cancer and thereby reduced their downstream signaling to promote cell proliferation and suppress cell apoptosis [20, 21]. In this study, we measured the miR-25 expression levels in the tissues of NSCLC patients and NSCLC cell lines. miR-25 was involved in NSCLC cell proliferation, migration, invasion, and xenograft tumor metastasis. Furthermore, we identified LATS2 as a new target gene for miR-25 in NSCLC metastasis. As miR-25 is induced by oxidative stress [22], our study provides profound insights into the oncogenic role of miR-25 and implicates this miRNA as a link between oxidative stress and metastasis and a promising therapeutic target to suppress metastasis of NSCLC.

## 2. Materials and Methods

### 2.1. Clinical Specimens

Between June 2017 and June 2018, we collected fifteen paired NSCLC tissues and their adjacent normal lung tissues from the Central Hospital of Wuhan. All tissue specimens were frozen in the liquid nitrogen immediately or stored in the refrigerator at -80°C after surgical removal. None of the patients accepted any chemotherapy or radiotherapy prior to tumor resection. All patients received informed consent during enrollment. This research was approved by the Ethical and Scientific Committees of the Central Hospital of Wuhan.

### 2.2. The Cancer Genome Atlas (TCGA) Analysis

Data on miR-25 expression and clinical information of NSCLC patients were obtained from TCGA data set. miRNAs-seq data were downloaded from TCGA website (cancergenome.nih.gov/), bcgsc.ca_LUAD.IlluminaHi-Seq_miRNASeq.Level_3.1.12.0, bcgsc.ca_LUSC.IlluminaGA_miRNASeq.Level_3.1.3.0, and bcgsc.ca_LUSC.IlluminaHiSeq_miRNASeq.Level_3.1.7.0. For survival analysis, we then stratified the patients into two groups, high and low expressions, according to miR-25 expression using the median of miRNA abundance as the threshold and then conducted a two-sample Kolmogorov-Smirnov test (K-S test) and draw the K-M plot.

### 2.3. Cells and Cell Culture

The NSCLC cell lines, including H1299, A549, H23, H520, and the normal lung epithelial cell line BEAS-2B, and human embryonic kidney (HEK) 293T cells were all purchased from American Type Culture Collection (ATCC) [21]. Lung carcinoma 95-D cell line was obtained from the Cell Bank of the Chinese Academy of Sciences [21]. All cells were cultured with suggested media in a 5% CO_2_ humidified incubator at 37°C as previously reported [21].

### 2.4. RNA Isolation and qRT-PCR

Total RNA was extracted from lung cancer tissues and cells using TRIzol reagent (Invitrogen, Carlsbad, CA, USA). The cDNA for target gene detection was synthesized from 1 *μ*g total RNAs. The target miRNA or mRNA was measured using SYBR Green (Applied Biosystems, Carlsbad, CA, USA) in the CFX96 real-time detection system (Bio-Rad, Hercules, CA, USA). U6 and *β*-actin were used as an endogenous control, respectively, for miR-25 and LATS2 detection. The miR-25 relative expression values were calculated using the 2^–*△*Ct^ method for lung cancer tissues or using the 2^–*△△*Ct^ method for cells. The primers for the detection of the LATS2 mRNA level were as follows: sense, TCC TGC CAC GAC TTA TTC, and antisense, GTG CCC GAT TCA TTA GC. LATS2 mRNA relative values were calculated by using the 2^–*△△*Ct^ method.

### 2.5. Transient Transfection of pSIF-GFP-miR-25 or miRNA Inhibitors

The pSIF-GFP-miR-25 precursor plasmid and the precursor control were gifts from professor Yong Li in Cleveland Clinic [20]. The transfection efficiency (>30%) of pSIF-GFP-miR-25 was checked based on GFP expression 24 h posttransfection by using a fluorescence microscope as reported in the previous study [21]. The miR-25 inhibitor and the negative control were purchased from GenePharm (Shanghai, China). The pSIF-GFP-miR-25 and the miR-25 inhibitor was used with a final concentration of 100 nM. The day before transfection, cells were plated to ensure ~70% cell confluence. Cell transfection was performed using Lipofectamine® LTX and Plus reagent (Invitrogen) following the manufacturer's protocol.

### 2.6. Colony Formation Assay

Cells were seeded into a 12-well plate (500 cells/well) and incubated for about ten days until colonies were apparent. The plate was then gently washed and stained with 0.1% crystal violet (Beyotime, Nantong, China). Colonies containing at least 50 cells were counted to observe the malignant viability of the single cell.

### 2.7. Wound Healing Assay

For cell mobility assay, cells were seeded onto 12-well plates (2 × 10^5^ cells/well); 24 h after transfection, an artificial wound was created by scratching the confluent cell monolayer using a sterile 200 *μ*l pipette tip. Then, cells were washed twice with phosphate-buffered saline (PBS) and incubated in RPMI-1640 supplemented with 1% FBS. The wound healing images (magnification, ×100) were taken at 0 h and 48 h after scratching. The wound healing rate was calculated by using the ImageJ software.

### 2.8. Invasion Assay

To investigate lung cancer cell invasion, transwell chamber (Corning, NY, USA) coated with Matrigel (BD Biosciences, CA, USA) was used. Twenty-four hours after transfection, cells were collected and suspended in 100 *μ*l serum-free medium. A total of 2 × 10^4^ cells were seeded into the upper chamber, and the complete culture medium was added into the lower chamber. After incubation for 48 h, cells on the upper surface of the membrane were removed using cotton swabs. Cells located on the lower filter surfaces were fixed in 4% paraformaldehyde solution (Beyotime) and stained with 0.1% crystal violet (Beyotime). The number of invading cells was determined by evaluating 5 fields per membrane using an IX81 microscope (Olympus, Tokyo, Japan). Images were taken at magnification 100x.

### 2.9. Luciferase Reporter Assay

The *LATS2* 3′UTR containing the putative miR-25 recognition elements was amplified (sense, 5′-TAT CTA GAG GAC TCA GCA TCG CTT TCA AT-3′, and antisense, 5′-ATG CGG CCG CTC ACA GCC ACA TCA TCA CCT T-3′). The mutated *LATS2* 3′UTR was also amplified (sense, 5′-TAT CTA GAG GAC TCA GCA TCG CTT TCA AT-3′, and antisense, 5′-ATG CGG CCG CTT ACA TTC GCT ACG AGA GAT TTC-3′). The wild-type and mutated PCR products were respectively subcloned into the pRL-TK vector (Promega). Correct constructs were confirmed by sequencing. Luciferase reporter assays were carried out in HEK-293T, A549, and H1299 cells as previously described [21].

### 2.10. Western Blot

Western blot analysis was conducted as previously described [23]. The primary antibodies were used as follows: rabbit polyclonal anti-LATS2 (1 : 500, Proteintech, Rosemont, USA), rabbit polyclonal anti-YAP (1 : 500, Proteintech), rabbit monoclonal anti-phospho-YAP (Ser127, 1 : 1000, Cell Signaling Technology, Danvers, MA, USA), rabbit monoclonal anti-E-cadherin (1 : 1000, Cell Signaling Technology), rabbit monoclonal anti-Vimentin (1 : 1000, Cell Signaling Technology), and rabbit polyclonal anti-MMP9 (1 : 500, Proteintech). Mouse monoclonal anti-*β*-actin (1 : 1000, Sigma-Aldrich) was used as a reference.

### 2.11. LATS2 Overexpression

The pClneoMyc-LATS2 plasmid was a gift from Yutaka Hata (Addgene plasmid), which expressed Myc-tagged LATS2 in mammalian cells [24]. A549 cells were seeded in a 12-well plate (2 × 10^5^ cells/well) and transfected with 2 *μ*g pSIF-GFP-miR-25 and/or 0.5 *μ*g pClneoMyc-LATS2 for 24 h. Transfected cells were then used for colony formation, wound healing, and invasion assays as described above.

### 2.12. Immunofluorescence Assay

For immunofluorescence assay, A549 cells were collected 48 h posttransfection and then seeded as 2 × 10^3^ cells/well in eight-well chamber slide (Millipore, Darmstadt, Germany). After incubation for 24 h, cells were washed with PBS and fixed with -20°C precold methanol for 10 min at -20°C. Then, cells were washed with PBS and incubated in 0.2% Triton for 10 min at room temperature. Cells were blocked in 5% fetal bovine serum for 1 h at room temperature and incubated with rabbit polyclonal anti-YAP (1 : 50, Proteintech) over night at 4°C and shaking. Next, cells were washed with PBS and incubated in a secondary antibody for 45 min. At last, cells were washed and stained with DAPI for 10 min. The images were taken by using a fluorescence microscope (Olympus BX51, Tokyo, Japan). ImageJ software was used to calculate the colocalization rate.

### 2.13. In Vivo Assay

The miR-25 antagomir/control miR-Down™ was obtained from GenePharm (Shanghai, China) [21]. Five-week-old BALB/c nude mice were purchased from HFK Bio-Technology Co. Ltd. (Beijing, China). A total of 5 × 10^6^ A549 cells, resuspended in Opti-MEM, were subcutaneously injected into the right flank of 6-week-old BALB/c nude mice (*n* = 5 for each group). The subsequent tumorigenesis experiments were performed as in the previous study [21]. For the tumor metastasis assay, a total of 2 × 10^6^ A549 cells in OptiMEM were injected into the tail veins of nude mice (*n* = 10 for each group). One week later, the miR-25 antagomir or control (200 *μ*l, 375 *μ*g per mice) was injected into the tail veins once per week for 3 weeks. One month after the first injection of miR-25 antagomir or control, mice were sacrificed and lungs and livers were collected for further analyses.

### 2.14. Statistical Analysis

Independent *t*-test, paired *t*-test, and *χ*^2^ test were carried out for statistical analysis using the SPSS 22.0 software. All experiments were repeated at least three times independently. *P* values less than 0.05 were considered to be statistically significant. ^∗^*P* < 0.05, ^∗∗^*P* < 0.01, and ^∗∗∗^*P* < 0.001.

## 3. Results

### 3.1. miR-25 Was Overexpressed in Both NSCLC Tissues and Cell Lines

**Figure 1:**
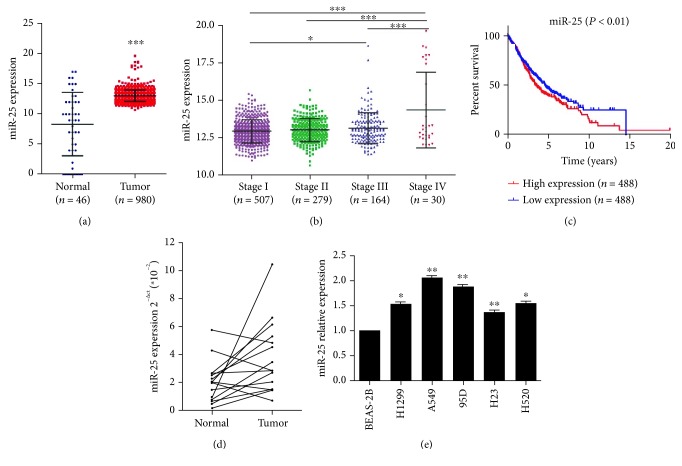
The expression of miR-25 in lung cancer tissues and cell lines. (a) miR-25 expression in the tumor tissues of NSCLC patients as compared to the control, noncancerous lung tissues from the TCGA database. (b) miR-25 expression in tumor tissues of NSCLC patients with different stages from the TCGA database. (c) In the TCGA database, the overall survival rate curves of NSCLC patients with high and low expression of miR-25 were shown. (d) The expression levels of miR-25 in 15 pairs of NSCLC tissues and the adjacent normal tissues by qRT-PCR. (e) The expression levels of miR-25 in NSCLC cell lines and the noncancerous lung epithelial cell line BEAS-2B. Results were presented as the mean ± SD. ^∗^*P* < 0.05, ^∗∗^*P* < 0.01, and ^∗∗∗^*P* < 0.001.

To explore the role of miR-25 in NSCLC, we analyzed miR-25 expression in NSCLC patients in the TCGA database. As shown in Figure 1(a), miR-25 was significantly elevated in NSCLC tissues (*n* = 980) compared with the control, noncancerous lung tissues (*n* = 46). Moreover, miR-25 was gradually increased with NSCLC stages I to IV (Figure 1(b)). The levels of miR-25 were significantly higher in patients with stage IV than those with stage I, stage II, and stage III. There was also significant difference in miR-25 expression between the patients with stages I and III. Next, NSCLC patients (*n* = 488) with a high expression of miR-25 presented worse overall survival than the patients (*n* = 488) with a low expression of miR-25 (Figure 1(c)). We further detected the expression of miR-25 in 15 pairs of paired NSCLC tissues and their adjacent normal lung tissues. The results showed that miR-25 was significantly upregulated in NSCLC tissues compared with their respective adjacent noncancerous tissues (Figure 1(d)). However, miR-25 was increased in 11/15 samples, suggesting that the upregulation of miR-25 occurred in approx. 2/3 of lung cancers, but not all (Figure 1(d)). Besides, qRT-PCR was used to evaluate the expression levels of miR-25 in various NSCLC cell lines and the normal lung epithelial cell line BEAS-2B. The results indicated that compared with noncancerous BEAS-2B cells, all five NSCLC cell lines expressed miR-25 at a higher level (Figure 1(e)). Because miR-25 expression was highest in metastatic cell line A549 and H1299 was a p53-null cell line which represents high malignancy, we chose these two lines as cell models in subsequent experiments.

### 3.2. miR-25 Enhanced NSCLC Cell Malignant Viability, Migration, and Invasion

**Figure 2:**
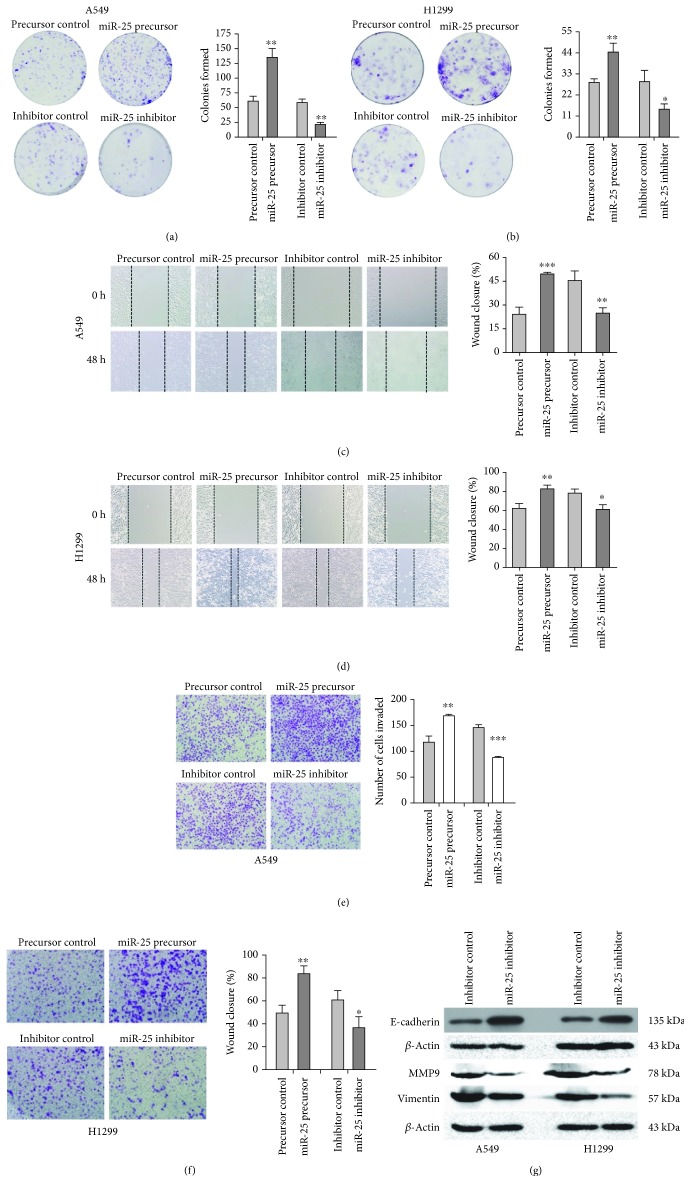
Overexpression or inhibition of miR-25 on cell proliferation, migration, and invasion in both A549 and H1299 cells. (a, b) Colony formation of cells transfected with the miR-25 precursor or the miR-25 inhibitor or with respective controls. (c, d) Wound healing assays for cell migration with altered miR-25 expression. (e, f) Transwell assays for cell invasion with altered miR-25 expression. (g) Western blot analyses of the expression of metastasis-related proteins, E-cadherin, Vimentin, and MMP9 in A549 and H1299 cells with altered miR-25 expression. Results were presented as mean ± SD of three independent experiments. ^∗^*P* < 0.05, ^∗∗^*P* < 0.01, and ^∗∗∗^*P* < 0.001.

The malignant viability of NSCLC cells was evaluated by a colony formation assay. When transfected with the miR-25 precursor, there were much more colonies formed by A549 (*n* = 124 ± 8) and H1299 (*n* = 44 ± 4) cells than the controls, A549 (*n* = 58 ± 7) and H1299 (*n* = 29 ± 2) cells transfected with the precursor control (Figures 2(a) and 2(b)). Adversely, the numbers of colonies were markedly decreased in A549 (*n* = 22 ± 4) and H1299 (*n* = 13 ± 3) when the miR-25 inhibitor was introduced, compared with those in A549 (*n* = 59 ± 6) and H1299 (*n* = 29 ± 6) with the inhibitor control (Figures 2(a) and 2(b)). Next, the migration and invasion ability of NSCLC cells were assessed by wound healing and transwell assays. There was a significant higher wound healing rate in A549 cells with the miR-25 precursor (wound closure is 49.47 ± 1.00%) than those with the control (wound closure is 29.50 ± 3.99%; Figure 2(c)). Similarly, transfection with the miR-25 precursor in H1299 cells also resulted in an increase in the wound healing rate (wound closure is 82.70 ± 4.42%) in comparison with the control (wound closure is 62.63 ± 4.63%; Figure 2(d)). Inhibition of miR-25 decreased the wound closure rates in both A549 and H1299 (Figures 2(c) and 2(d)). For the transwell assay, as shown in Figure 2(e), the invaded cell number increased from 118 ± 6 to 170 ± 3 in A549 cells with the miR-25 precursor, whereas the invaded cell number decreased from 146 ± 5 to 88 ± 2 in A549 cells with the miR-25 inhibitor. Similar cell invasion effects were also observed when miR-25 was inhibited or overexpressed in H1299 cells (Figure 2(f)). The above results suggest that miR-25 enhanced lung cancer malignant viability, migration, and invasion. We further detected protein markers of cancer metastasis in A549 and H1299 cells. As shown in Figure 2(g), following the treatment of the miR-25 inhibitor, the level of the epithelial marker E-cadherin was upregulated, while the levels of the mesenchymal marker Vimentin and the cell matrix metalloproteinase MMP9 was downregulated in A549 and H1299 cells. These results indicate that the inhibition of miR-25 may suppress EMT and extracellular matrix degradation in NSCLC.

### 3.3. *LATS2* Is a Direct Target of miR-25

**Figure 3:**
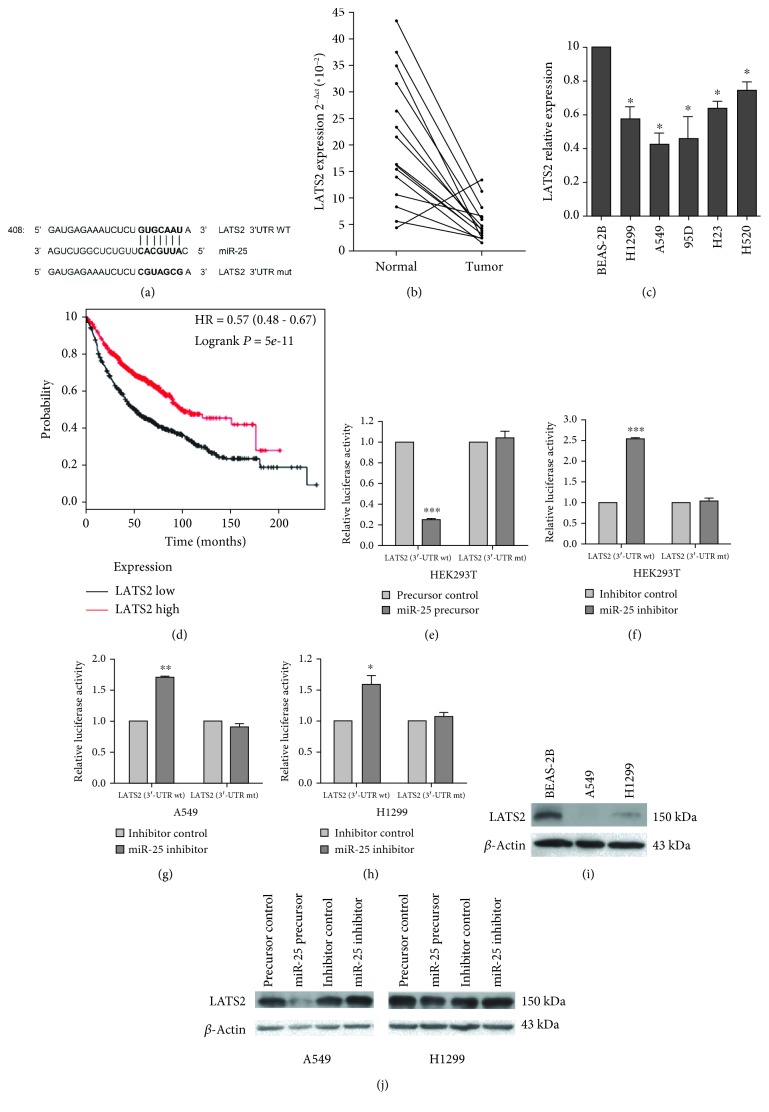
LATS2 is a target gene of miR-25 [21]. (a) The predicted binding site of miR-25 in the 3′UTR of *LATS2* from miRBase. The mutated *LATS2* 3′UTR-binding site is indicated. (b) LATS2 expression in 15 pairs of NSCLC tissues and adjacent normal tissues by qRT-PCR. (c) LATS2 expression in NSCLC cell lines and the noncancerous lung epithelial cell line BEAS-2B. (d) Kaplan-Meier curve for overall survival in lung cancer patients with high or low LATS2 expression. Data were taken from TCGA. (e, f) Luciferase reporter assays in HEK-293 cells. (g, h) Luciferase reporter assays in H1299 cells. HEK-293T, A549, and H1299 cells were cotransfected with pRL-TK carrying a wild-type or mutant 3′UTR of LATS2 and the miR-25 precursor (60 ng) or the miR-25 inhibitor (10 pmol), and the luciferase activity was measured 48 h posttransfection. (i) Western blot assays for LATS2 in A549, H1299, and BEAS-2B cells. (j) Western blot analysis of LATS2 in A549 and H1299 cells transfected with miR-25 precursor or the miR-25 inhibitor. Experiments in this section were performed using our documented protocols [21]. Results were presented as the mean ± SD. ^∗^*P* < 0.05 and ^∗∗∗^*P* < 0.001.

Among the predicted targets of miR-25 in miRBase (version 21), *LATS2* located at chromosome 13q11-12 encodes the LATS2 protein, a Ser/Thr kinase regulating cell biological processes and a key component in the Hippo pathway. *LATS2* is reported as a target gene of miR-25 in ovarian cancer [25] and gastric cancer [26], yet such a relationship in lung cancer has not been established. There is a putative binding site for miR-25 in the 3′UTR of *LATS2* (Figure 3(a)). We used established procedures to determine whether LATS2 is an authentic miR-25 target gene [21]. We first examined LATS2 mRNA expression in 15 pairs of lung cancer tissues and their adjacent normal tissues by qRT-PCR. We found that LATS2 levels were lower in lung cancer tissues than in adjacent normal tissues (Figure 3(b)). In addition, LATS2 was reduced in NSCLC cell lines as compared to the noncancerous lung epithelial cell line BEAS-2B (Figure 3(c)). Data from the Cancer Genome Atlas (TCGA) indicated that lung cancer patients (*n* = 573) with a high expression of LATS2 exerted higher overall survival rates than those (*n* = 572) with a low expression of LATS2 (Figure 3(d)), suggesting a tumor suppressive role for LATS2 in lung cancer [21].

Next, we conducted luciferase reporter assays in HEK293T and H1299 cells by placing either the wild-type LATS2 3′UTR or a mutant lacking miR-25 binding sites (Figure 3(a)) downstream of the luciferase gene. We found that when miR-25 was upregulated, luciferase activities were decreased in cells carrying the luciferase gene with the wild-type 3′UTR, but not in those with the mutant 3′UTR (Figure 3(e)). Conversely, when miR-25 was downregulated, there was a significant increase in luciferase activities in HEK293T, A549, and H1299 cells with the wild-type 3′UTR but not in those with the mutant 3′UTR (Figures 3(f)–3(h)). We noted that the LATS2 protein level was lower in both A549 and H1299 cells than in BEAS-2B cells (Figure 3(i)). When miR-25 was overexpressed, LATS2 protein levels were significantly downregulated in A549 and H1299 cells; and LATS2 protein levels were elevated when miR-25 was suppressed (Figure 3(j)).

### 3.4. miR-25 Promotes NSCLC Cell Malignant Viability, Migration, and Invasion by Targeting LATS2

**Figure 4:**
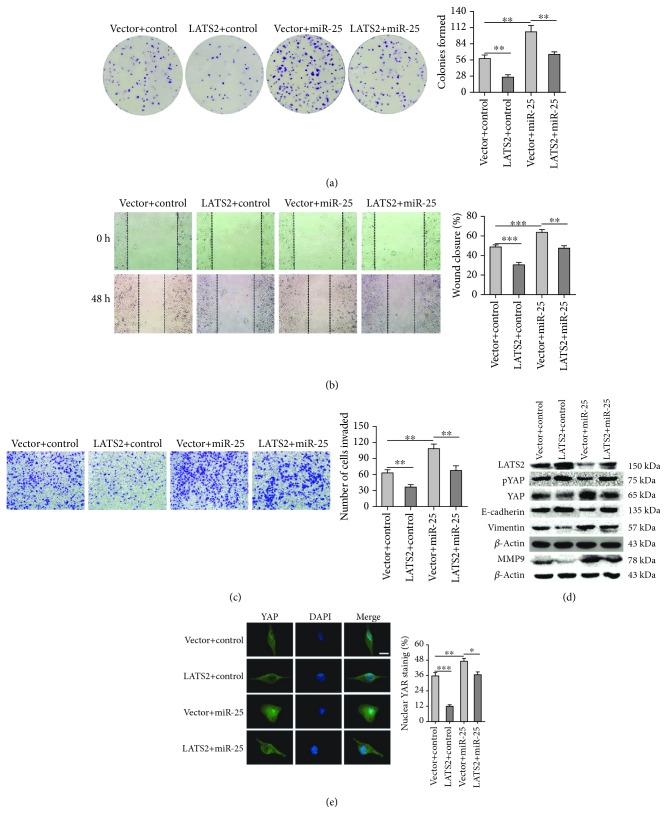
The effects of LATS2 expression on A549 cell proliferation, migration, and invasion. (a) Colony formation of A549 cells transfected with LATS2, miR-25, or the combination of both miR-25 and LATS2 plasmids. (b, c) Wound healing and transwell assays for migration and invasion capacities of A549 cells transfected with miR-25 and/or LATS2. (d) Western blot assays for LATS2, downstream effectors of the Hippo pathway (phosphorylated YAP and YAP), and metastasis-related proteins (E-cadherin, Vimentin, and MMP9) in A549 cells transfected with miR-25 and/or LATS2. Results were presented as the mean ± SD. (e) Immunofluorescence assay for nuclear translocation of YAP protein. Representative immunofluorescent images of YAP staining were shown in the left panel (green: YAP; blue: nuclear; scale bar, 10 *μ*m). The nuclear localization of YAP is quantified in the right panel by ImageJ software. ^∗^*P* < 0.05, ^∗∗^*P* < 0.01, and ^∗∗∗^*P* < 0.001.

To further explore whether miR-25 regulates NSCLC cell proliferation and metastasis by targeting LATS2, we transfected A549 cells with pClneoMyc-LATS2 or the miR-25 precursor and evaluated the malignant phenotypes. As shown in Figure 4(a), the formed colonies of A549 cells was reduced from 58 ± 6 to 27 ± 3 following LATS2 overexpression, while they were elevated from 58 ± 6 to 105 ± 11 when miR-25 was overexpressed. Yet, the overexpression of LATS2 reversed the positive effects of miR-25 overexpression-mediated colony formation. As expected, the overexpression of LATS2 markedly inhibited the wound closure rate and decreased the cell invasion of A549 cells, while overexpression of miR-25 exhibited the opposite effects (Figures 4(b) and 4(c)). Similarly, the miR-25-mediated enhanced migration and invasion of A549 cells were reversed by cotransfection with LATS2 (Figures 4(b) and 4(c)). These results suggest that miR-25 promotes NSCLC cell malignant viability, migration, and invasion, at least partially, by targeting LATS2.

As a key serine/threonine kinase in the Hippo signaling pathway, LATS2 phosphorylates the Hippo downstream effector YAP oncoprotein, reduces the level of YAP, and inhibits its shuttling from the cytoplasm to the nucleus, to exert its tumor-suppressive effects [27]. We detected the LATS2-related proteins in the Hippo pathway: YAP and phosphorylated YAP. As shown in Figure 4(d), when LATS2 was overexpressed, the protein levels of phosphorylated YAP and E-cadherin were elevated, whereas YAP, Vimentin, and MMP9 were downregulated. Overexpression of miR-25 had opposite effects, including decreased LATS2 expression, suppressed YAP phosphorylation and E-cadherin, and increased YAP, Vimentin, and MMP9 levels. The impact of miR-25 overexpression was partially reversed by cooverexpression of LATS2. Further, immunofluorescent staining was used to analysis the nuclear accumulation of YAP in A549 cells. As shown in Figure 4(e), LATS2 overexpression inhibited the nuclear accumulation of YAP, whereas miR-25 overexpression promoted YAP protein nuclear localization. The miR-25-mediated enhanced nuclear accumulation of YAP in A549 cells was reversed by cotransfection with LATS2. These data support that LATS2 is a *bona fide* target of miR-25.

### 3.5. miR-25 Antagomir Inhibits Lung Tumor Growth and Metastasis In Vivo

**Figure 5:**
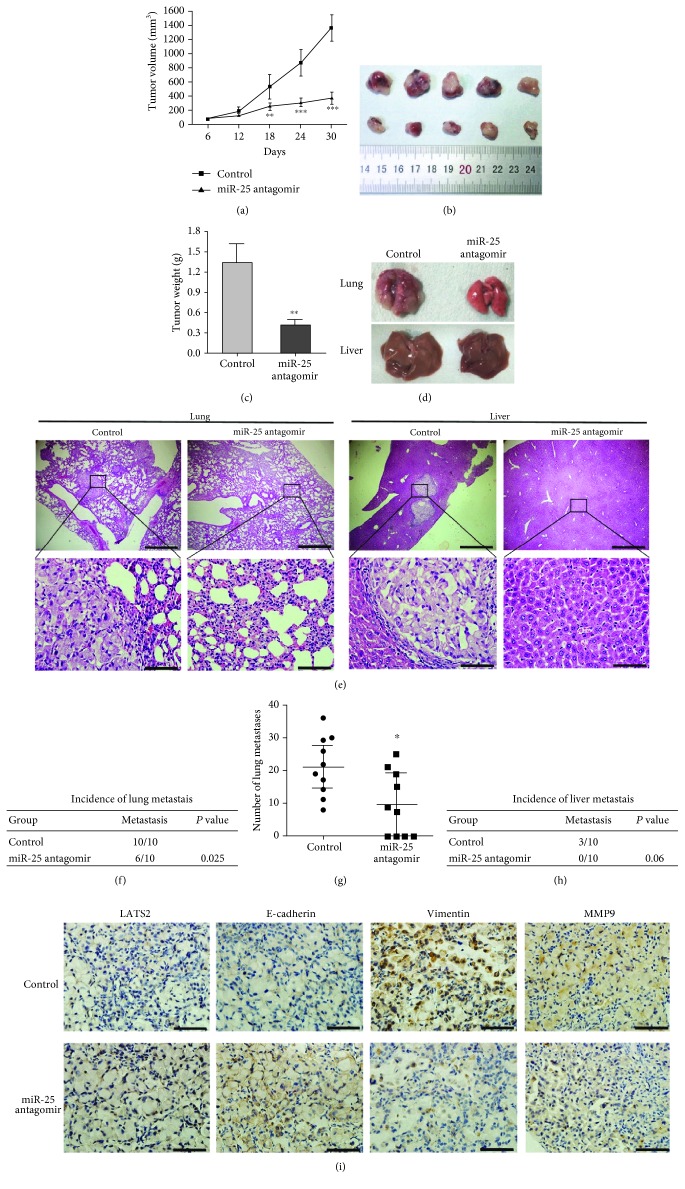
miR-25 antagomir inhibits lung cancer growth and metastasis in mouse xenografts. (a) Time course of tumor volumes from immunodeficient mice treated with the miR-25 antagomir or a control. Results were presented as the mean ± SD. ^∗∗^*P* < 0.01 and ^∗∗∗^*P* < 0.001. (b, c) The image and the weight of xenograft tumors one month after the treatment. Mean ± SD was shown. ^∗∗^*P* < 0.01. (d) Representative images of mouse lungs and livers. (e) Histological morphology of mouse lungs and livers by HE staining. The magnification of the upper row images was 40x, bar scale = 500 *μ*m. The magnification of the bottom row images was 400x, bar scale = 50 *μ*m. (f, h) The incidence of lung and liver metastasis in mice after tail intravenous injections with the miR-25 antagomir was shown in the table. (g) Quantification of the metastasis nodules in the lungs of each group (*n* = 10). ^∗^*P* < 0.05. (i) Representative images of immunohistochemical analysis of LATS2, E-cadherin, Vimentin, and MMP9 in lung metastasis (magnification, 400x. bar scale = 50 *μ*m). Brown represented the target protein in IHC, and blue represented nuclear staining.

To determine whether the inhibition of miR-25 affects lung tumorigenesis in vivo, we injected the miR-25 antagomir and control intratumorally when the tumors xenografted with A549 cells reached an average of 60 mm^3^ in immunodeficient mice. We found that tumors injected with the miR-25 antagomir exhibited slower growth than those with the control (Figure 5(a)). One month after the first dose of antagomir, tumor size (Figure 5(b)) and weight (Figure 5(c)) were significantly decreased. Next, we investigated whether the inhibition of miR-25 reduces lung cancer cell metastasis in vivo by injecting A549 cells through the tail vein. One week later, mice received the miR-25 antagomir or control. We examined the lungs and livers of the host mice one month after antagomir treatment (Figure 5(d)). As shown in Figures 5(e), 5(f), and 5(h), lung metastases were found in 100% of mice and liver metastases in 30% of mice in the control group; in the miR-25 antagomir group, 60% of lung metastases and 0% of liver metastases were observed. In addition, the 60% of lung metastases are micro metastases. The lung histology indicated that the number of metastases in the antagomir group was smaller than the control (Figure 5(g)). In addition, immunohistochemistry analysis showed that the expression of LATS2 and E-cadherin was noticeably higher, while that of Vimentin and MMP9 was lower in the lung metastases from mice treated with the miR-25 antagomir than from the control mice (Figure 5(i)). These results implicate that the inhibition of miR-25 significantly reduces tumor growth and metastasis of lung cancer by elevating LATS2 expression in vivo.

## 4. Discussion

Lung cancer is a major cause of cancer death worldwide [28]. Metastasis, for the most part, is responsible for the high mortality of lung cancer, underlying the urgency to find novel therapeutic targets for metastasis. Given that miRNAs are frequently dysregulated in cancer, more and more researchers are investigating oncogenic or tumor-suppressive miRNAs in lung cancer. The expression of miR-25, miR-26a, miR-127, and miR-134/miR-487b/miR-655 cluster was upregulated in lung cancer cells, and knockdown of these miRNAs suppressed cell proliferation and invasion [29–32]. For miR-218, miR-335, miR-200c, and miR-194 that were downregulated in lung cancer, their overexpression resulted in the reduced cell growth, migration, and invasion [33–36].

A previous report demonstrates that miR-25 is significantly upregulated by oxidative stress [22], supporting miR-25 as a potential drug target for antioxidative therapy. Our studies indicated that miR-25 was upregulated in the plasma of lung cancer patients and it promoted cell proliferation and inhibited apoptosis in lung cancer cells by targeting *TP53* and *MOAP1* [20, 21]. In the present study, we found that miR-25 expression was elevated in both the tissues of NSCLC patients and lung cancer cell lines. NSCLC patients with higher expression of miR-25 exhibited worse overall survival than those with lower expression of miR-25. Furthermore, the overexpression of miR-25 in A549 and H1299 cells enhanced cell viability, migration, and invasion, while the inhibition of miR-25 showed the opposite effects. Our results, in combination with previous studies, imply that miR-25 is an important biomarker for lung cancer diagnosis and a promising drug target for lung cancer metastasis.

To investigate the underlying mechanisms of miR-25 in enhancing lung cancer cell metastasis, we identified *LATS2* as a novel target of miR-25. LATS2 plays a critical role in the Hippo signaling pathway [37]. Accumulating evidence supports that the LATS family of human tumor suppressors (LATS1 and LATS2) is a new governor of cell homeostasis [38] and LATS2 is downregulated in many cancers, such as prostate cancer [39] and colon cancer [40]. We showed that LATS2 was downregulated in tumor tissues of NSCLC patients and lung cancer cell lines, in agreement with a previous report [41]. As the mRNA levels of LATS2 and miR-25 expression were reversed in NSCLC, we further investigated the regulatory mechanism involved in them. As expected, a luciferase reporter assay and western blot analysis identified that *LATS2* was a direct target gene of miR-25 in lung cancer. Overexpression of LATS2 reversed the effects of miR-25 on cell proliferation, migration, and invasion. More importantly, the downstream effectors of LATS2 in the Hippo signaling pathway, YAP, phosphorylated YAP, E-cadherin, Vimentin, and MMP9 were all altered by modulating the expression of miR-25 and/or LATS2. Meanwhile, the enhanced nuclear accumulation of YAP by miR-25 overexpression in A549 cells was reversed by cotransfection with LATS2. In addition, the in vivo mouse xenograft experiments demonstrated that the inhibition of miR-25 suppressed tumor growth and lung cancer metastasis. Moreover, immunohistochemical analysis suggested that the slower tumor growth rate and the reduced metastasis by miR-25 inhibition were, at least in part, due to the upregulation of LATS2 and E-cadherin expression and the downregulation of Vimentin and MMP9. Collectively, these data support that miR-25 is an important regulator of the key kinase LATS2 in the Hippo pathway and provided new insights into the upstream regulation of Hippo signaling in lung cancer.

**Figure 6:**
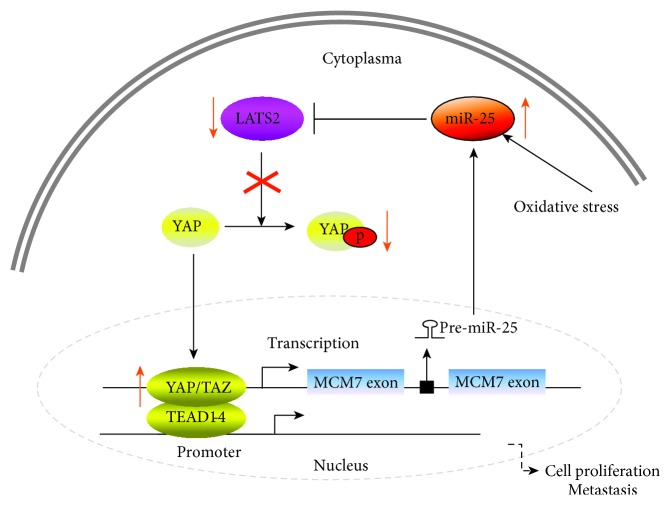
Schematic presentation of a positive feedback loop involving miR-25/LATS2/YAP/TAZ/TEAD/*MCM7*/miR-25 in lung cancer.

Previous reports showed that the host gene of miR-25, *MCM7*, was upregulated upon YAP or TAZ overexpression in MCF10A cells [42, 43]. *MCM7* was found to be a target of the Hippo pathway transcriptional coactivators YAP/TAZ by ChIP-seq in breast cancer cells [44]. In line with these results, YAP directly bound to the *MCM7* enhancer in NSCLC cells and elicited the oncogenic role of YAP/TAZ through modulation of a bioncogenic locus consisting of *MCM7* and its three hosted miRNAs [45]. In our study, we identified that LATS2 was a direct target of miR-25 in lung cancer. Given that LATS2 is an upstream regulator of YAP in the Hippo pathway, we summarize a positive feedback loop pathway in NSCLC (Figure 6); miR-25 overexpression, induced by oxidative stress in NSCLC, inhibits LATS2 expression, which reduces the phosphorylation of YAP, promoting the translocation of YAP oncoprotein from the cytoplasm to the nucleus. YAP/TAZ cooperates with the TEAD cotranscriptional activators in the nucleus and binds to a distal enhancer around the MCM7 promoter. Upregulated MCM7 and the miR-106b-25 cluster, in turn, inhibit the expression of LATS2. Additionally, excessive YAP/TAZ/TEAD cotranscriptional activators in the nucleus promote the transcription of genes involved in cell proliferation and metastasis. This positive feedback loop underscores the oncogenic role of miR-25 in promoting lung cancer cell proliferation and metastasis and may explain why miR-25 is upregulated in lung cancer.

Taken together, the present study demonstrate the oncogenic role of miR-25 in lung cancer. Oxidative stress-induced miR-25 overexpression promotes cell proliferation, migration, and invasion in NSCLC by directly inhibiting LATS2 and subsequently increasing downstream protein YAP nuclear translocation, constituting a positive feedback loop to regulate NSCLC metastasis. These findings implicate miR-25 as a promising therapeutic target for lung cancer metastasis.

## Data Availability

The data used to support the findings of this study are available from the corresponding authors upon request.
